# Ketogenic Diet-Induced Diabetic Ketoacidosis in a Young Adult with Unrecognized Type 1 Diabetes

**DOI:** 10.1155/2021/6620832

**Published:** 2021-02-08

**Authors:** Suranut Charoensri, Jin Sothornwit, Akeatit Trirattanapikul, Chatlert Pongchaiyakul

**Affiliations:** ^1^Division of Endocrinology and Metabolism, Department of Medicine, Faculty of Medicine, Khon Kaen University, Khon Kaen, Thailand; ^2^Department of Medicine, Faculty of Medicine, Khon Kaen University, Khon Kaen, Thailand

## Abstract

Ketogenic diet, a very low-carbohydrate diet and high-fat diet, has emerged as a popular approach for weight reduction, particularly in young adults. However, a serious but rare complication of the ketogenic diet is ketoacidosis associated with low carbohydrate intake, which should be cautiously monitored in people with a predisposition to the condition. We report a 22-year-old Thai woman with an unremarkable past medical history who presented with an acute onset of dyspnea of 2 days' duration. Diabetic ketoacidosis was diagnosed by elevated capillary blood glucose, significant metabolic acidosis, and a high serum beta-hydroxybutyrate level. Low C-peptide level and positive islet autoantibodies confirmed the new diagnosis of type 1 diabetes in this patient. After her conditions were stabilized, the patient revealed that she began a ketogenic diet for weight reduction 4 days before her illness. Other precipitating factors were not identified. This highlights that ketogenic diet may increase diabetic ketoacidosis risk at the presentation of previously unrecognized type 1 diabetes.

## 1. Introduction

A number of dietary patterns have been proposed as an effective method for weight reduction [1, 2]. Guideline recommendations support several weight-loss approaches that focus on the alteration of macronutrients, including low-fat, low-carbohydrate, and high-protein diets [3]. All of these approaches have resulted in similar clinically meaningful weight loss [4, 5]. The ketogenic diet, a very low-carbohydrate diet and high-fat diet, has emerged as a popular approach, especially for young adults. This diet restricts the daily intake of carbohydrates to less than 50 grams per day, leading to reduced insulin secretion and the conversion of fatty acids to ketone bodies for energy consumption [6, 7]. However, a serious but rare complication of the ketogenic diet is ketoacidosis associated with low carbohydrate intake [8], which should be cautiously monitored in people with a predisposition to the condition, including pregnancy, chronic alcoholism, and type 1 diabetes [9]. Here, we report the case of a healthy young adult who developed diabetic ketoacidosis (DKA) as the first presentation of type 1 diabetes following the course of ketogenic diet.

## 2. Case Report

A 22-year-old Thai woman with an unremarkable past medical history presented to the emergency department with an acute onset of dyspnea of 2 days' duration. She was unable to tolerate any food or drink since the symptoms started. On examination, her vital signs included temperature 38°C, pulse rate 110 bpm, blood pressure 110/80 mmHg, and respiratory rate 32/minute with a kussmaul breathing pattern. Other physical examinations were unremarkable except for severe dehydration. The initial testing revealed the results in Table 1, which included capillary blood glucose of 356 mg/dL, serum bicarbonate of 4.8 mEq/L, arterial pH of 7.15, and a serum beta-hydroxybutyrate level of 6.65 mmol/L, while the lactate level was normal. The urinalysis showed glucosuria and ketonuria. A diagnosis of new-onset diabetes and diabetic ketoacidosis (DKA) was established. However, the type of diabetes was undetermined at that time. Fasting C-peptide level and islet autoantibodies were sent to evaluate for type 1 diabetes.

She was admitted to the intensive care unit and treated with intravenous saline, intravenous insulin infusion, and electrolyte repletion. Blood glucose levels and metabolic acidosis improved. Oral intake and subcutaneous insulin were initiated two days later. Despite a BMI of 22.3 kg/m^2^, the patient felt that she needed to lose weight. A week prior, she initiated an intensive weight management regimen including a ketogenic diet. She had restricted her carbohydrate intake to less than 15 grams per day 4 days. The specifics of her ketogenic meal are shown in Table 2. She denied participating in a heavy exercise which could lead to dehydration. Other precipitating factors of DKA were not identified.

After 6 days of hospital admission, she was discharged with multiple daily injection (MDI) insulin therapy without further complications. One week later, the results of special investigations for identifying the type of diabetes showed C-peptide level 0.2 ng/mL, anti-GAD 8.79 U/mL (normal <5), and anti-IA2 130.95 U/mL (normal <7.5), which confirmed the diagnosis of type 1 diabetes in this patient.

## 3. Discussion

This case demonstrates the development of DKA following a low-carbohydrate, ketogenic diet in a patient with unrecognized type 1 diabetes. It is important for physicians to be aware of this rare potential complication of ketogenic diet, particularly in those with increased risk for type 1 diabetes.

DKA is a life-threatening complication, usually affecting children with newly diagnosed type 1 diabetes [10, 11]. Although the mortality rate is low, DKA is associated with adverse neurological outcomes [12]. The most common precipitating factors in the development of DKA are infection and discontinuation of insulin in type 1 diabetes [13]. Factors that may lead to insulin omission in young patients include fear of weight gain, fear of hypoglycemia, rebelliousness from authority, and stress of chronic disease [14]. Theoretically, carbohydrate-restricted dietary ketosis may predispose to ketoacidosis in people with type 1 diabetes, particularly in the setting of insulin omission or in the rare situation of undiagnosed type 1 diabetes, not on insulin therapy [9].

Adolescent obesity is a global epidemic with a growing prevalence impacting all socioeconomic groups [15]. However, perceptions of overweight in late adolescence and early adulthood are much more common than being actual overweight. Up to 60% of normal weight young women consider themselves to be overweight in both Western and Asian cultures [16, 17]. These beliefs, whether true or not, are correlated with unhealthy habits, including maladaptive weight-loss strategies [18, 19]. Ketogenic diet is one of the most popular weight-reduction methods in Thailand. Ketosis status from this form of dietary pattern is predicted. However, severe ketoacidosis inducing metabolic acidosis is rare [20]. In patients with near-normal or normal insulin activity, the concentration of plasma beta-hydroxybutyrate during a ketogenic diet should be comparable to those who fasted because the rate of hepatic ketone body synthesis is balanced by ketone utilization and ketone excretion in the urine [9, 21–23]. Therefore, there will be no alteration of the acid-base balance during dieting. On the contrary, serious ketoacidosis may occur in people with lower than normal insulin activity, including type 1 diabetes, pregnant women, alcoholics, and certain diabetic patients currently using sodium-glucose cotransporter-2 inhibitors (SGLT-2i). These patients with higher risk for ketoacidosis should be monitored more closely if choosing to pursue a ketogenic diet. The patient reported in our case had an unrecognized insulin deficiency, combined with a strict ketogenic diet of less than 15 grams of carbohydrates per day, which led to a presentation of DKA. It is important to make individuals aware of this potential complication. In those at higher risk for type 1 diabetes, especially in first-degree relatives of type 1 diabetes patients or other high-risk populations, clinicians should consider screening for overt diabetes prior to patients starting a ketogenic diet, and in those with type 1 diabetes on ketogenic diets, insulin omission should be avoided.

## Figures and Tables

**Table 1:** Initial laboratory investigations.

Lab	Value	Reference	Units
Hemoglobin	13.7	12.0–14.3	g/dL
Hematocrit	41	36.0–47.7	%
White blood cells	15.8	4.60–10.60	k/mm^3^
Neutrophils	82	43.7–70.9	%
Platelets	294	173–383	k/mm^3^
Sodium	144	136–145	mEq/L
Potassium	3.6	3.5–5.0	mEq/L
Chloride	115	98–106	mEq/L
Bicarbonate	4.8	23–30	mEq/L
Anion gap	24.2	3–11	—
BUN	3.8	6.0–20.0	mg/dL
Creatinine	0.49	0.51–0.95	mg/dL
Glucose	390	70–99	mg/dL
Hemoglobin A1C	14	4.6–6.5	%
Lactate	1.2	0.4–2.0	mmol/L
Serum ketones	6.65	0–3	mmol/L
Urine glucose	4+	Negative	—
Urine ketones	2+	Negative	—
Arterial pH	7.15	7.35–7.45	—

**Table 2:** The “ketogenic diet” that the patient had before her illness.

Day	Lunch	Dinner
Day 1	(i) One serving of Thai papaya salad	(i) Eight servings of spiced duck
	(ii) Half serving of fried fish	(ii) One cup of black coffee
	(iii) A coconut	

Day 2	(i) One serving of Thai papaya salad	(i) One serving of stir-fried pork with basil leaves (without rice)
	(ii) Half serving of fried fish	(ii) One serving of omelette
	(iii) One cup of black coffee	(iii) A coconut
	(iv) One bottle of soda	(iv) One dish of fried pupa

Day 3	(i) One serving of Chinese clear soup with braised pork	(i) A tablespoon of avocado
	(ii) A coconut	(ii) 30 grams of pumpkin seed
	(iii) One cup of latte	

Day 4	(i) One serving of Chinese clear soup with braised pork	(i) One serving of Thai suki

## Data Availability

The data used to support the findings of this study are available from the corresponding author upon request.
